# Media attitudes toward wolves reflect recolonization phases, livestock predation peaks, and electoral cycles

**DOI:** 10.1007/s13280-026-02408-9

**Published:** 2026-04-26

**Authors:** Davide Ravaglia, Guillaume Chapron, Francesca Marucco

**Affiliations:** 1 Carnivore and Ungulate Research for Conservation Laboratory, Department of Life Sciences and Systems Biology, University of Torino, Via Accademia Albertina, 13, 10123 Turin, TO Italy; 2 Department of Ecology, Swedish University of Agricultural Sciences, Grimsö Wildlife Research Station, Riddarhyttan, Sweden

**Keywords:** *Canis lupus*, Human dimensions, Human–wildlife conflict, Large carnivore conservation, Media attitudes, Political elections

## Abstract

The ongoing recolonization of Europe by wolves (*Canis lupus*) has generated substantial societal attention reflected in media narratives, where attitudes vary in their framing of conservation success and human–wildlife conflict. We examined factors influencing media attitudes toward wolves by analyzing over 4000 online news articles from the Italian Alpine regions across a decade, employing both human and neural network classifications. Bayesian modeling identified clear spatial and temporal patterns: negative media sentiment prevailed in recently recolonized areas, whereas positive attitudes emerged in regions with established wolf populations and at the national level. Negative sentiment correlated strongly with seasonal peaks in predation on livestock and proximity to regional, national, and European elections. We revealed how spatial recolonization dynamics, human–wildlife conflicts, and electoral cycles collectively shape media framing of a recolonizing controversial carnivore. These findings inform conservation strategies that address human–wildlife conflict dynamics while acknowledging the politicized dimension surrounding the species.

**Graphical abstract:**

**Figure:**
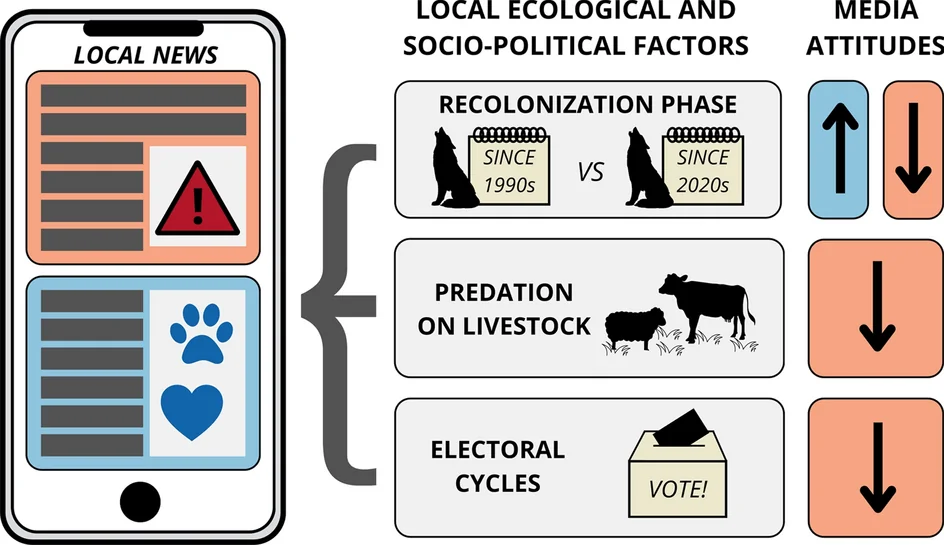

**Supplementary Information:**

The online version contains supplementary material available at https://doi.org/10.1007/s13280-026-02408-9.

## Introduction

Large carnivores have been recovering across Europe after centuries of persecution (Chapron et al. 2014; Di Bernardi et al. 2025). This conservation success has sparked public interest and drawn considerable media attention, particularly regarding the wolf (*Canis lupus*) (Dressel et al. 2015). Because large carnivores are elusive and maintain relatively low population densities, direct human encounters remain uncommon; consequently, media coverage of the species has been linked to public attitudes (Enck and Brown 2002), making the media a significant stakeholder in shaping conservation policies (Fernández-Gil et al. 2016). Previous research has documented that both human and media attitudes toward wolves vary across recolonization phases, from species absence to recent recolonization to longer coexistence, though results are inconsistent about which phase is associated with more negative attitudes (Dressel et al. 2015; Barmoen et al. 2024), and some clarity might be achieved with more localized analysis. For media attitudes specifically, recently recolonized areas can exhibit more negative coverage (Houston et al. 2010), mirroring patterns observed for human attitudes (Zimmermann et al. 2001). This novelty effect, in which unfamiliarity with wolf presence can amplify negative framings that later recede as local communities gain direct experience with the species and its real impacts, may overshadow the impact of increased wolf density in established areas. This is consistent with evidence that media coverage does not reliably reflect actual conflict rates, as demonstrated by Fernández-Gil et al. when comparing media coverage of wolf damages and actual costs of wolf damages (Fernández-Gil et al. 2016).

Being a species of ecological importance and societal concern, wolves carry political significance as other large carnivores do, and the media serves as a key forum for expressing and debating political opinions about them. In the USA, wolf management debates have been shown to center on symbolism and underlying values, with political events reshaping discourse (Wheeler et al. 2025) and management preferences regarding large carnivores being associated with political affiliation (Van Eeden et al. 2021). Similarly, in Europe, anti-wolf stances are common among politicians representing rural areas, who leverage such positions as symbols for broader political agendas to appeal to constituencies they might otherwise neglect (Chapron and López-Bao 2014; Clemm von Hohenberg and Hager 2022). This politicization can emerge during electoral campaigns, when anxieties about rural livelihoods and elite-driven environmental policies focus on wolves as tangible targets (Darimont et al. 2018). While media attitudes are likely influenced by multiple factors (e.g., media political orientation and regional contexts), understanding how concrete indicators of wolf presence, such as predation on livestock or recolonization dynamics and politicization, are linked to media discourse can inform the timing and targeting of conservation efforts. We undertook a multidisciplinary, large-scale, and long-term investigation: over a decade, we monitored both the expansion of the wolf population in the Italian Alpine regions and how the media framed these events.

Wolf recovery in this area began more than 30 years ago through natural dispersal from the Apennines, establishing first in the Western Alps (Fabbri et al. 2007). Eastward expansion followed more than a decade later, initiated by stochastic long-distance dispersal events and subsequent recolonization of adjacent areas (Marucco et al. 2023). Throughout this process, wolves progressively recolonized human-populated landscapes, resulting in the diverse recolonization phases observed across Italian Alpine regions today, which correspond to documented variation in public attitudes toward wolves (Bele et al. 2022). This mosaic of recolonization histories, ranging from decades-long presence to recent arrivals, provides a natural framework for comparing how recolonization phases relate to media narratives and calls for close monitoring to assess potential associations with negative opinions about wolf recovery, heightened risk of illegal killings (Liberg et al. 2012; Frank 2016), and management interventions influenced by political considerations rather than empirical evidence (Darimont et al. 2018).

We analyzed over 4000 wolf-related news articles published in 2014–2023, covering different recolonization phases across the Alpine region. We employed Bayesian hierarchical modeling to test three hypotheses: (I) media attitudes are spatially and temporally structured by wolf recolonization patterns, with expectations of the most negative attitudes in recently recolonized areas where novelty on human–wildlife conflict could influence coverage before nuanced understanding develops (Zimmermann et al. 2001; Houston et al. 2010); (II) media attitudes are more negative during periods of peak predation on livestock, one of the main drivers of conflict (Niemiec et al. 2020; Gervasi et al. 2022); and (III) media attitudes shift in proximity to elections, when wolves can be exploited as political symbols (Chapron and López-Bao 2014; Darimont et al. 2018; Clemm von Hohenberg and Hager 2022). Our goal was to understand how recolonization dynamics, human–wildlife conflicts, and political events collectively shape media attitudes toward large carnivores.

## Materials and methods

### Sampling design of data collection and media attitude assessment

**Fig. 1:**
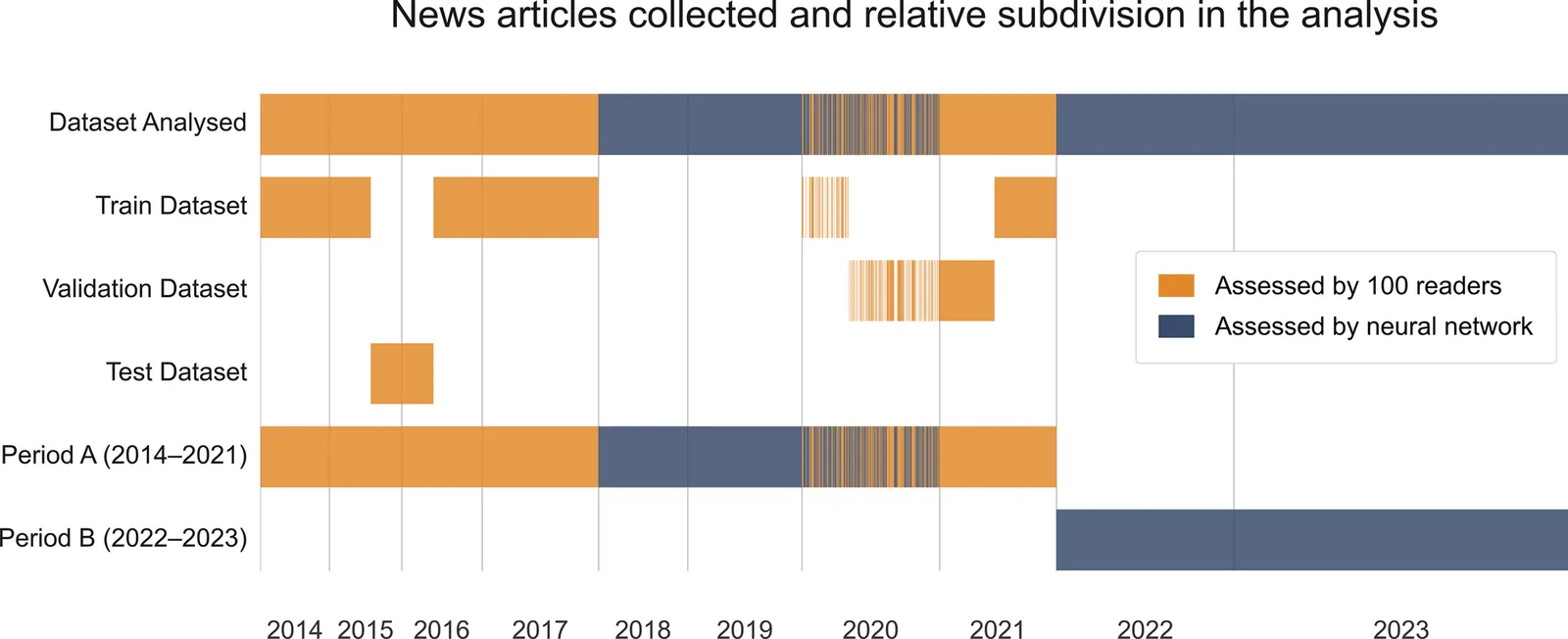
Representation of news articles in the different datasets sorted temporally by publication date. Articles assessed by the 100 readers (2014–2017 and 2020–2021) are shown in orange, while articles classified by the neural network (2018–2019 and 2022–2023) are shown in steel blue. The first row presents the totality of the news articles assessed by the readers and the neural network. The second to fourth rows illustrate how we divided the readers-assessed subset into training, validation, and test sets to develop the neural network. The fifth and sixth rows show how we partitioned the news articles into two datasets to highlight the recent recolonization process: Period A (2014–2021) and Period B (2022–2023). Additional details about the dataset are provided in the Supplementary Information

The news article collection was conducted by a team of coordinated researchers from 2014 to 2024 using Google News Alerts and Searches, consistently applying the Italian keyword “wolf”. Articles were collected continuously by the media office of the LIFE Wolfalps (2014–2017) and LIFE Wolfalps EU (2020–2021) projects, with a dedicated team that ranged from 1 to 3 observers depending on the availability of human resources and project funding. To ensure continuous coverage, an additional dedicated researcher collected data in April 2024 for periods not covered by the two projects (2018–2019, parts of 2020, and 2022–2023) using the same sampling strategy and alignment criteria ensuring consistency across the collection period (Supplementary Information, Page 1). This process ultimately compiled a total of 4094 news articles from nearly 200 sources, representing the breadth of Italian media coverage on this topic over 10 years. Each article was geographically referenced based on the province or region of the news outlet’s reach. Additionally, we gathered data on election dates occurring at various administrative scales, including European, national, and regional elections, from the publicly available database *Eligendo Archivio* (elezionistorico.interno.gov.it). The resulting dataset comprised two subsets (Fig. 1): articles collected within the LIFE project periods (*n* = 1622), assessed by a pool of human readers mobilized within the project framework, and articles collected outside these periods (*n* = 2472), assessed by a neural network trained on the human-evaluated subset to extend sentiment classification beyond the project periods.

Articles from the LIFE project periods were distributed to the readers based on a stratified design controlling for demographic factors such as sex, age, self-reported species knowledge, and pre-existing attitudes toward wolves, with approval by the Bioethics Committee of the University of Turin. Each article’s attitude toward wolves was thus evaluated independently by four different readers, allowing for a measure of concordance between readers (Supplementary Information, Table S1), using a 5-point Likert scale ranging from “extremely negative” (− 2) to “extremely positive” (+ 2). Individual media articles may contain multiple viewpoints and complex narratives about wolves. While this single-scale approach necessarily reduced such complexity, it provided a consistent and comparable measure of overall tone in media discourse.

Articles collected outside the LIFE project periods were assessed using a neural network based on the BERT architecture (Devlin et al. 2019). The neural network was trained on articles collected within the LIFE projects, supplemented with 156 articles from printed press archives^1^ collected to increase early-year coverage and assessed by the 100 readers. These articles were later excluded from the main analysis to maintain consistency with the Google News-based collection strategy and retained solely to broaden neural network training exposure. The neural network generated four assessments per article on the identical Likert scale employed by the human evaluators. These four assessments were obtained by training the network multiple times on a subset of the 100-reader articles (Fig. 1), producing varied predictions analogous to inter-reader variability (Supplementary Information, Page 3). We validated this approach on held-out subsets of the 100-reader articles (Fig. 1): automated classifications obtained were more aligned with the mode of the readers’ assessments than any two random readers were with one another, while maintaining a comparable distribution of attitudes (Supplementary Information, Table S1). While automated media analysis has been employed in wildlife studies through structural topic modeling (Chandelier et al. 2018; Braun et al. 2025), BERT applications are novel in this field, though validated for sentiment analysis across diverse datasets (Bello et al. 2023). Following state-of-the-art guidelines (Sun et al. 2019), our implementation demonstrates that such innovative methods enable large-scale longitudinal monitoring of media attitudes.

**Fig. 2:**
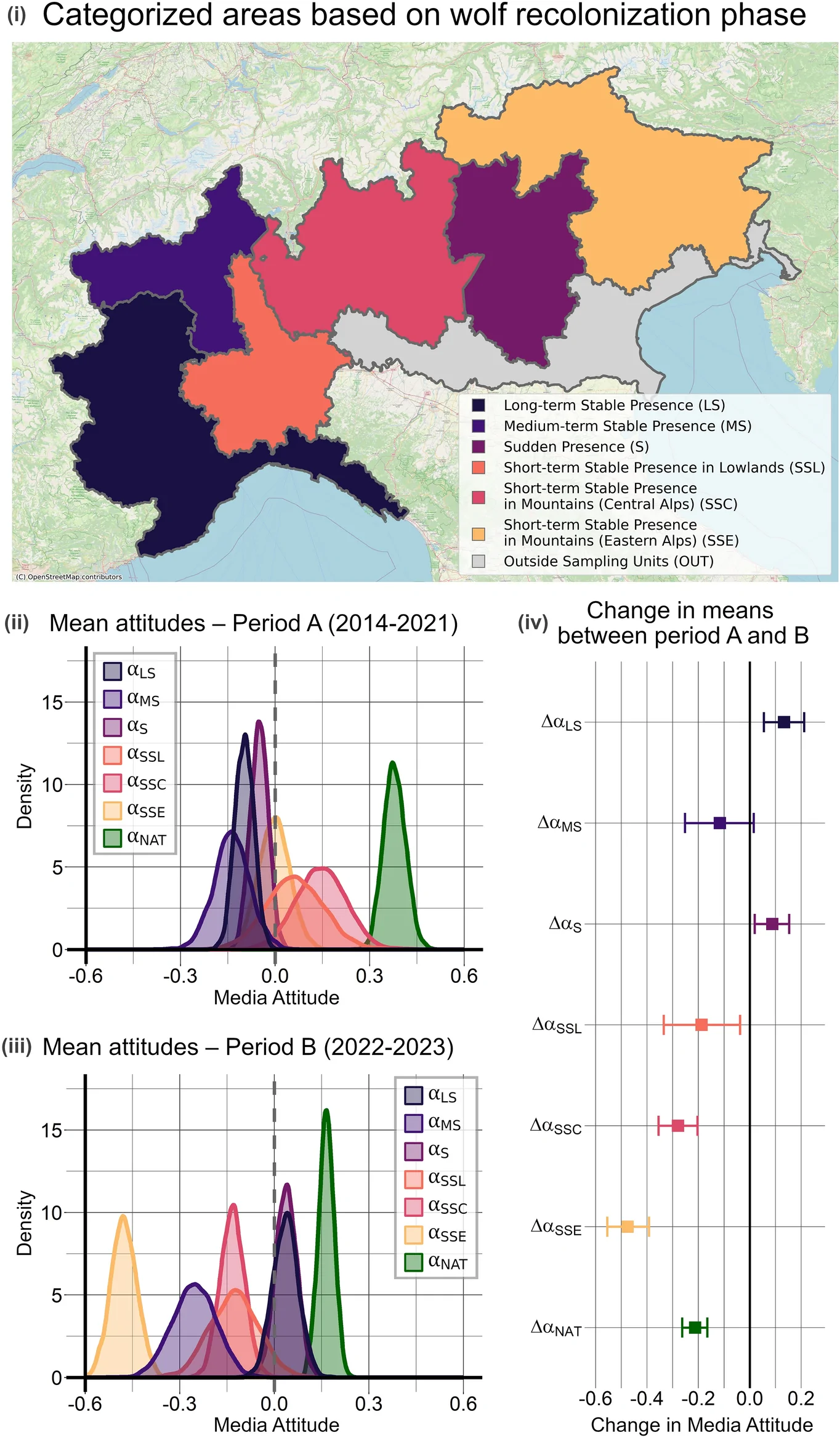
The subdivision of the Italian Alpine regions into six categorized areas of different wolf recolonization phases, illustrating how mean media attitudes shifted between the time intervals, Period A and Period B. At the top (*i*), the map indicates the six categorized areas based on the phase of wolf recolonization, as defined in the legend. The bottom-left panel (*ii* and *iii*) displays the posterior distributions of mean media attitudes for the six categorized areas and the national level in green (NAT), both for Period A (2014–2021) and Period B (2022–2023). The bottom-right panel (*iv*) depicts the differences between Period B median estimate and Period A 95% Credible Interval, revealing a negative shift in recently recolonized areas (α_SSL_, α_SSC_, and α_SSE_)

We categorized the recolonization phases at the provincial scale across the Italian Alpine regions based on 25 years of recolonization dynamics and on the relative wolf pack density regularly documented over the years by Marucco et al. (2023), identifying six distinct areas arranged based on recolonization history (Fig. 2):

1. *Long-term stable presence* (LS): Wolf packs gradually recolonized since 1990s and now occur at high density, with humans and media exposed to wolves for approximately 30 years.
2. *Medium-term stable presence* (MS): Wolf packs gradually recolonized since 2005 and now occur at moderate density, with humans and media exposed to wolves for approximately 15 years.
3. *Sudden presence* (S): Wolf packs rapidly recolonized following a stochastic event in 2012 and now occur at high density, with humans and media suddenly exposed to wolves for over 10 years.
4. *Short-term stable presence in lowlands* (SSL): Wolf packs recolonized human-dominated urbanized lowland provinces since 2020s and now occur at low density, with humans and media exposed to wolves only recently.
5. *Short-term stable presence in mountains (central alps)* (SSC): Wolf packs recolonized mountain provinces since 2020s, following recolonization of neighboring areas, and now occur at low density, with humans and media exposed to wolves only recently.
6. *Short-term stable presence in mountains (Eastern Alps)* (SSE): Wolf packs recolonized mountain provinces since 2020s following sudden recolonization of neighboring areas and now occur at low density, with humans and media exposed to wolves only recently.

News articles were spatially assigned to these categorized areas (Fig. 2) based on the geographic reach of their publication. Articles with geographic reach outside these areas were categorized separately as OUT, while articles with a national level focus were designated as NAT.

### Bayesian model

Our dataset presents two modeling challenges: Media attitudes are recorded on an ordinal Likert scale yet arise from a continuous underlying sentiment, and recolonization timelines differ across regions, requiring area-specific estimates within a shared analytical framework. A hierarchical Bayesian ordinal regression addresses both, recovering the latent metric process behind ordinal responses while estimating region-level parameters informed by the overall dataset. We assumed an underlying continuous metric variable representing media attitudes toward wolves to avoid analyzing ordinal data as metric (Liddell and Kruschke 2018), an approach recently applied also to attitudes toward large carnivores across Europe (Chapron et al. 2025). The Likert scale divides this continuous variable through unevenly spaced thresholds *τ*_*k*|*k*+1_ that separate two consecutive media attitude classes *k* and *k* + 1 (Kruschke 2014). We further assumed that this metric variable follows a normal distribution characterized by a mean (*μ*) and a standard deviation (*σ*) (Kruschke 2014). We developed a Bayesian hierarchical ordinal regression with the thresholds *τ*_−2|−1_ and *τ*_+1|+2_ fixed, respectively, at − 1.5 and 1.5, testing whether four drivers linked to our hypothesis affected the mean. First, we estimated different means α_X_ for each spatial category, where X represents the spatial attribution LS, MS, S, SSL, SSC, SSE, NAT, or OUT previously defined. Second, we incorporated a linear trend dependent on Year_*i*_, the year of article publication, estimating its slope (*β*). Third, we included a sinusoidal intra-annual trend based on the day of publication (day_*i*_), estimating amplitude (*γ*_Ampl_), and phase in days (*γ*_Days_) of the sinusoid, designed to capture seasonal fluctuations in media attitudes associated with the livestock grazing season and the corresponding peak in predation events. Fourth, we accounted for election proximity by creating a predictor based on an exponential function of days until the next relevant election (elect_*i*_) and a half-life parameter that established the time frame of election interest (T = 180 days, informed by a sensitivity analysis across values from 30 to 360 days; Supplementary Information, Fig. S4). We then multiplied this predictor by an amplitude parameter ψ, estimating both the magnitude and direction (positive or negative) of the election proximity effect. Considering these four factors, we computed the mean attitude *μ*_*i*_ as:

$${\mu }_{i}=\sum_{X\in \mathrm{Areas}}\left({\alpha }_{X} \cdot {\theta }_{X,i}\right)+\beta \cdot {\mathrm{Year}}_{i}+{\gamma }_{\mathrm{Ampl}} \cdot \mathrm{cos}\left(\frac{2\pi }{365.25} \cdot ({\mathrm{day}}_{i}-{\gamma }_{\mathrm{Days}})\right)+\Psi \cdot (0.5{)}^{{elect}_{i} / T}$$

where θ_*X*,*i*_ are binary indicator variables equal to 1 for the *X* label corresponding to the article’s spatial attribution and 0 otherwise. We implemented the model in R using JAGS (Kruschke 2014), applying Markov Chain Monte Carlo (MCMC) sampling with minimally informative priors to estimate posterior distributions of all parameters shown in bold in the equation. We then applied the Bayesian model to media attitude data collected from 2014 to 2023, subsequently partitioning the dataset into Period A (2014–2021) and Period B (2022–2023). This division was guided by the known timing of recent wolf recolonization (Marucco et al. 2023) and corroborated by a concurrent increase in media coverage (Fig. 1), which suggested that recolonization dynamics were influencing news production. This increase also meant that, despite spanning only 2 years, Period B provided a substantial number of articles for robust analysis. Given the limited number of events or reduced interpretability associated with certain parameters in the Period B dataset (e.g., election proximity and temporal trends), we fixed the parameters *β*, *γ*_Ampl_, *γ*_Days_, and ψ at values estimated from the complete 2014–2023 dataset for this latter analysis. We validated Bayesian model performance by recovering known parameter values from simulated article‐assessment datasets and by running posterior‐predictive checks on the 2014–2021, 2014–2023, and 2022–2023 datasets (Supplementary Information, Table S3 and Fig. S5). We also confirmed MCMC convergence after burn-in by inspecting the trace plots (Supplementary Information, Figs. S6–S8).

## Results

Local media attitudes reflect the phase of wolf recolonization: newly recolonized areas exhibited more negative media attitudes, whereas regions with a longer history of stable wolf presence displayed more positive media attitudes (Table 1, Fig. 2), supporting Hypothesis I. This contrast emerges when dividing the articles by year of publication into Period A (2014–2021) and Period B (2022–2023), during which wolf-related news rose substantially in the three recently recolonized areas (Supplementary Information, Fig. S1). This pattern emerged only in Period B, not in the earlier neural network-assessed period (2018–2020), indicating genuine temporal change linked to wolf population expansion. During Period A, three categorized areas without extended wolf presence showed positive mean media attitudes (α parameters, Table 1), whereas areas with stable wolf populations leaned negative (Fig. 2). By contrast, during Period B, media attitudes turned more negative in the three newly recolonized areas, whereas two historically recolonized areas shifted toward more positive attitudes (Table 1, Fig. 2). Indeed, comparing posterior distributions by subtracting the Period B median from Period A 95% Credible Interval (CI) shows only newly recolonized areas displayed significant negative change (Fig. 2), reinforcing Hypothesis I. National media attitude was more positive than local media attitude (Table 1, Fig. 2). We found little support for a linear trend over 2014–2023, as the common time trend (β parameter, Table 1) remained negligible.

**Table 1:** Three hypotheses (I–III) are tested through Bayesian model parameters. Wherever no 95% CI is shown, the parameter value was set to the 2014–2023 dataset estimate. The categorized area labels are defined as follows: Long-term Stable Presence (LS), Medium-term Stable Presence (MS), Sudden Presence (S), Short-term Stable Presence in Lowlands (SSL), Short-term Stable Presence in Mountains (Central Alps) (SSC), and Short-term Stable Presence in Mountains (Eastern Alps) (SSE)

Parameterized drivers of media attitudes						
Hypothesis	Parameters used to test the hypothesis			Median and 95% CI		
				2014–2023 entire dataset	2014–2021 Period A	2022–2023 Period B
*I:* media attitudes are spatially and temporally structured by wolf recolonization patterns, with expectations of the most negative attitudes in recently recolonized areas (test: *α*_SSL_, *α*_SSC_, *α*_SSE_ and become more negative in 2022–2023 and are the most negative α in that period)	Mean of media attitude	3 historically recolonized categorized areas	*α*_LS_	− 0.099 [− 0.146, − 0.053]	− 0.098 [− 0.155, − 0.039]	0.036 [− 0.043, 0.114]
			*α*_MS_	− 0.220 [− 0.309, − 0.131]	− 0.137 [− 0.250, − 0.025]	− 0.254 [− 0.389, − 0.122]
			*α*_*S*_	− 0.112 [− 0.159, − 0.066]	− 0.050 [− 0.106, 0.005]	0.037 [− 0.031, 0.103]
		3 recently recolonized categorized areas	*α*_SSL_	− 0.076 [− 0.197, 0.042]	0.063 [− 0.112, 0.242]	− 0.127 [− 0.274, 0.024]
			*α*_SSC_	− 0.068 [− 0.145, 0.011]	0.146 [− 0.008, 0.299]	− 0.133 [− 0.209, − 0.057]
			*α*_SSE_	− 0.292 [− 0.359, − 0.227]	− 0.003 [− 0.099, 0.093]	− 0.478 [− 0.558, − 0.396]
		National	*α*_NAT_	0.379 [0.314, 0.448]	0.233 [0.188, 0.278]	0.165 [0.114, 0.213]
	Time trend common to all categorized areas		*β*	− 0.0095 [− 0.0163, 0.0025]	− 0.0202 [− 0.0323, − 0.0077]	− 0.0095
*II:* media attitudes are more negative during periods of peak predation on livestock (test: *γ*_Ampl_ < 0 and *γ*_Days_ ∈ [165, 285])	Yearly media attitude cosine pattern	Amplitude	*γ*_Ampl_	− 0.206 [− 0.234, − 0.180]	− 0.291 [− 0.328, − 0.254]	− 0.206
		Phase in days	*γ*_Days_	234.3 [226.7, 242.0]	237.9 [230.0, 245.6]	234.3
*III:* media attitudes shift in proximity to elections (test: *ψ* ≠ 0)	Intensity of nonlinear trend linked to election proximity		*ψ*	− 0.114 [− 0.183, − 0.042]	− 0.449 [− 0.548, − 0.354]	− 0.114

**Fig. 3:**
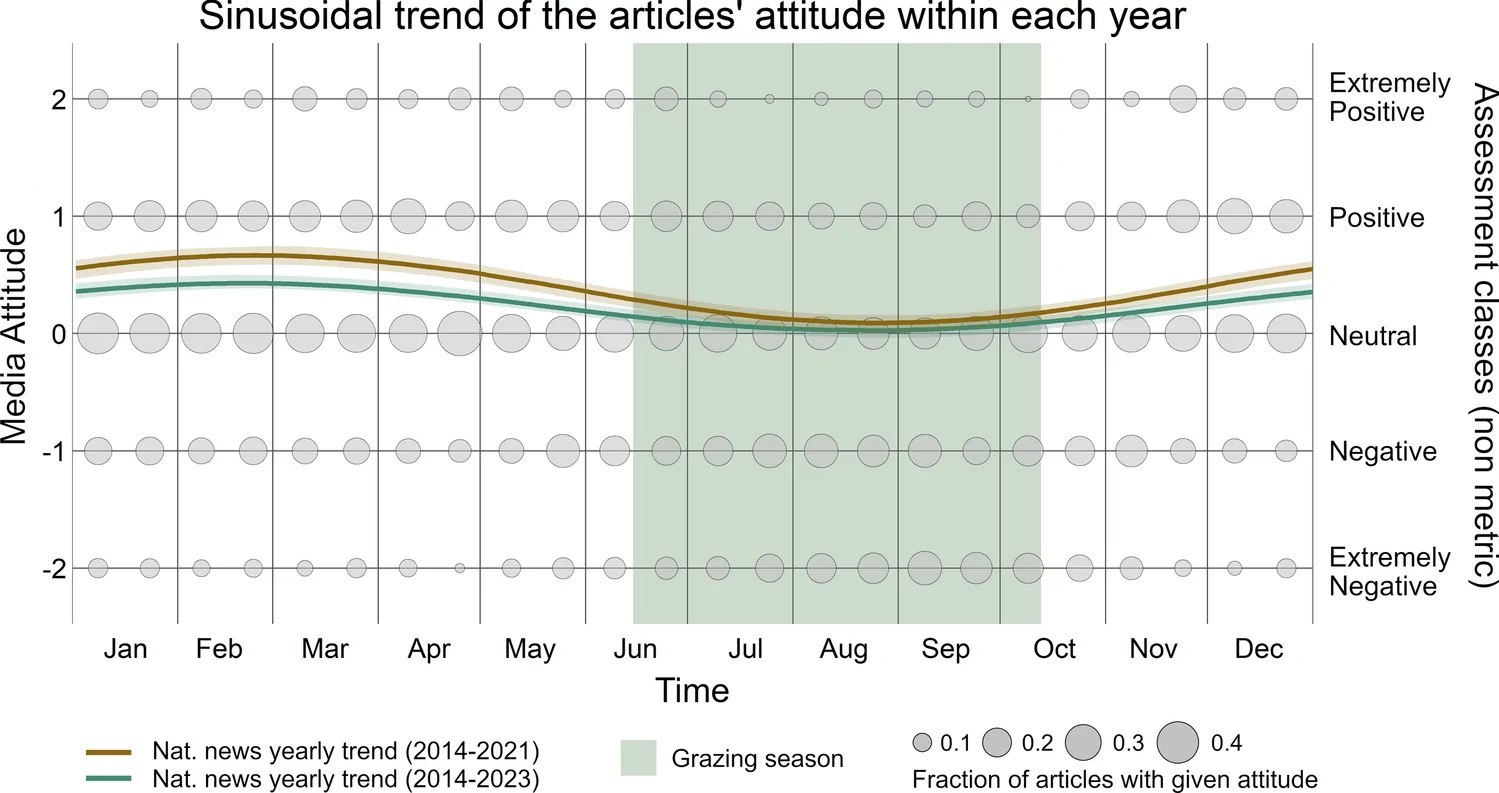
The intra-annual cosine pattern influencing mean media attitude, estimated from the 2014–2021 and 2014–2023 datasets and depicted for news at the national level. The two trend lines represent estimates from the two datasets: the golden line shows the 2014–2021 pattern and the green line the 2014–2023 pattern. The shaded bands surrounding each trend reflect uncertainty (100 samples drawn from the 95% credible intervals of the parameter posterior distributions). Gray circles represent empirical data: the size of each circle indicates the fraction of news article assessments across all spatial scales falling into each attitude class (ranging from extremely negative to extremely positive), grouped by publication date. The dull green band corresponds to the grazing season

We observed significant seasonal variation in media attitudes, marked by negative coverage during the grazing season, which is the time of peak predation on livestock, supporting Hypothesis II. To test this effect, we evaluated whether media attitudes fluctuate each year according to a wave-like pattern, which we modeled using a yearly cosine function characterized by an amplitude and phase. The amplitude’s 95% CI (γ_Ampl_ parameter, Table 1) was consistently negative, and the phase’s 95% CI (γ_Days_ parameter, Table 1) fell between days 230–245, indicating reduced mean media attitude in July–September, aligning with documented rises in predation on livestock (Gervasi et al. 2022). Figure 3 illustrates this pattern for national news, with increased “negative” and “extremely negative” articles during the grazing season. This effect translates into annual fluctuations of approximately 0.5 in mean media attitudes (double of the amplitude γ_Ampl_, Table 1), significant enough to encompass differences among categorized areas experiencing distinct recolonization phases.

**Fig. 4:**
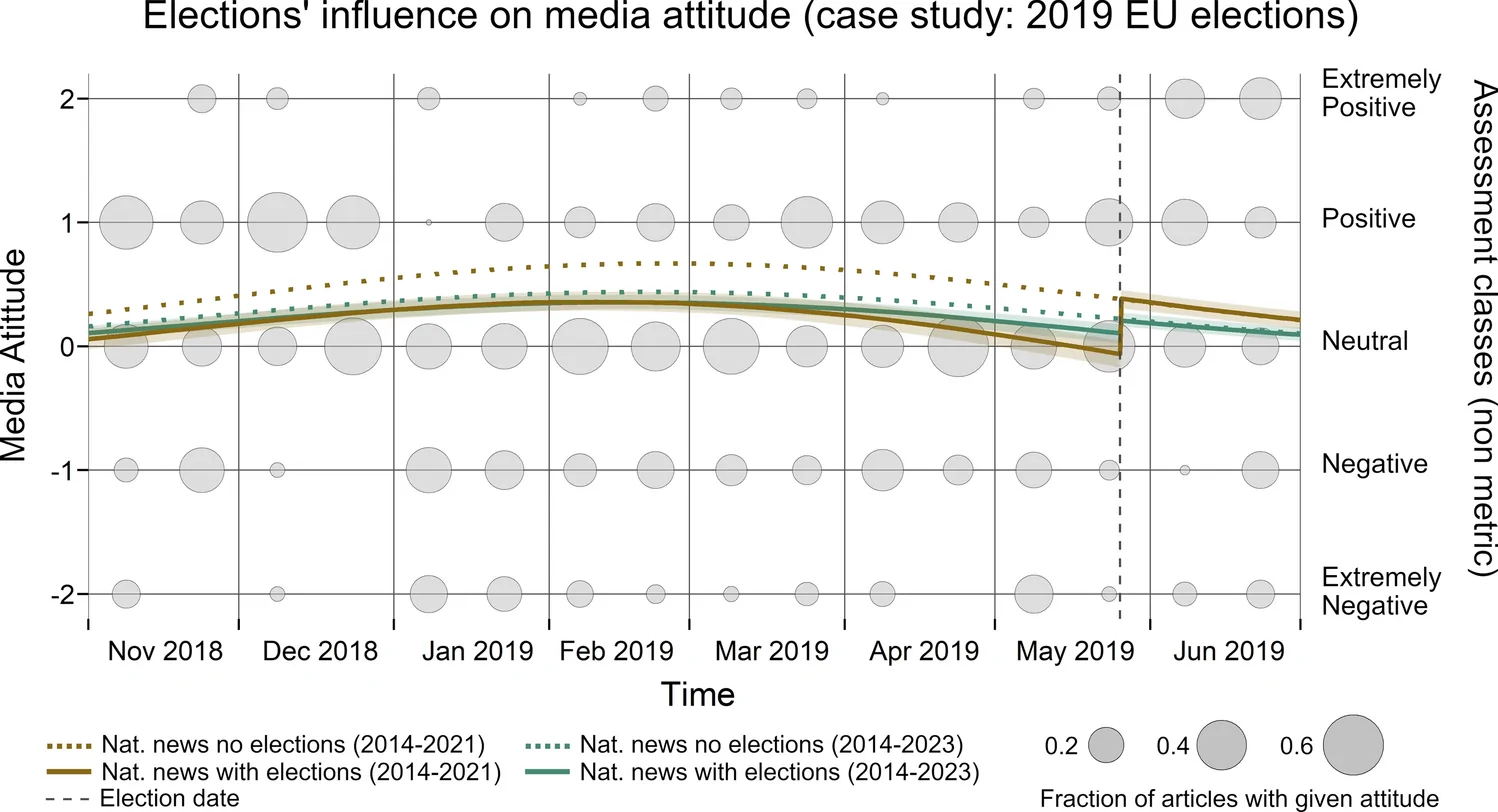
The negative impact of election proximity on mean media attitude, depicted for news at the national level, shown for the European general election of May 26, 2019. The effect is estimated from both the 2014–2021 and 2014–2023 datasets (golden and green trend lines, respectively). Dashed lines represent mean media attitude with only the sinusoidal effect linked to predation on livestock, while the solid lines incorporate the additional negative influence of the approaching election date. The shaded bands surrounding the trend lines reflect uncertainty (100 samples drawn from the 95% credible intervals of the parameter posterior distributions). Gray circles represent empirical data: The size of each circle indicates the fractions of news article assessments across all spatial scales falling into each attitude class (ranging from extremely negative to extremely positive), grouped by publication date

Once accounting for recolonization phases and seasonal variation, election proximity is associated with negative media attitudes, supporting Hypothesis III. As elections approached, mean media attitudes became increasingly negative. We measured this through the intensity (*ψ* parameter, Table 1) of a nonlinear trend, which was consistently negative. Specifically, for the 2014–2021 dataset (23 election dates), *ψ* was –0.449, while for the 2014–2023 dataset (30 election dates), *ψ* was − 0.114. These values show how, right before elections, media attitudes decrease, with this effect gradually fading as publication dates fall further from elections. Figure 4 illustrates this effect superimposed on the yearly sinusoidal trend for the 2019 European elections.

## Discussion

Wolf recolonization phases drive structured spatial and temporal variation in media attitudes, mirroring changes in wolf pack presence and stability. In line with Houston et al. (2010), our findings indicate increased negative coverage in recently recolonized areas with lower wolf pack densities (Marucco et al. 2023). This effect was present in both mountainous and lowland regions, regardless of the recolonization paths of the adjacent areas. This pattern likely reflects heightened novelty, uncertainty, and risk perception during early exposure to wolf presence (Delibes-Mateos 2020), when limited knowledge of the species can amplify the fear elicited by predation events (Barmoen et al. 2024). Local media outlets can amplify these initial tensions, focusing on incidents or threats rather than ecological or conservation perspectives (Killion et al. 2019). Indeed, these narratives attract greater public attention (Soroka et al. 2019) by aligning with prevailing concerns about safety and economic loss. For human attitudes toward wolves, spatial proximity with the species can reduce support (Karlsson and Sjöström 2007), though this effect can diminish once initial recolonization subsides (Zimmermann et al. 2001). Recent EU-wide surveys confirm that support for large carnivore recovery remains strong even in areas with established populations (Chapron et al. 2025), suggesting adaptation over time. Our results support this pattern, showing that two historically recolonized regions now exhibit the most positive average media attitudes despite higher wolf pack densities. This pattern is possibly due to the public gradually adapting to coexistence while management frameworks, compensation schemes, and knowledge of wolf behavior become more consolidated (Houston et al. 2010), though other unmeasured local population characteristics, such as rurality, knowledge, and attitudes toward the species, may also contribute. Thus, longer coexistence with the species, even if alongside higher wolf pack densities (Marucco et al. 2023), does not necessarily diminish media support, contrasting earlier trends reported in a meta-analysis conducted in Europe (Dressel et al. 2015). To detect recolonization phase effects, spatially structured analysis at fine scales is essential, as contrasting local responses average out when aggregated across regions with different wolf histories. Regardless of the recolonization phase, local media attitudes were consistently more negative than those at the national level. Although this disparity has diminished in recent years (Fig. 2), suggesting that local news in historically recolonized regions may eventually become more positive than national coverage, this relationship still holds. The stronger positivity in national media likely reflects both less emphasis on the local events that often trigger human–wildlife conflicts (Chandelier et al. 2018; Killion et al. 2019) and the unequal distribution of coexistence costs and benefits: Predation losses are borne locally while the broader ecological benefits of wolf recovery are shared across society at large (Jordan et al. 2020). Recognizing these local challenges, the proactive sharing of coexistence success stories could help foster more positive narratives (Braun et al. 2025), particularly in recently recolonized areas where negative framing may reflect initial uncertainty rather than intractable opposition, contributing to long-term tolerance.

We observed pronounced seasonal fluctuations in media attitudes during the grazing season, when predation on livestock peaks (Gervasi et al. 2022). This trend likely stems from coverage of predation events illustrated with attention-drawing images and titles (Bombieri et al. 2018). Although we lacked detailed data on specific predation frequencies, such responses to increased predation events are expected, given both the economic losses and the anger and fear elicited among stakeholders (Zahl-Thanem et al. 2020). The strong correlation between seasonal predation peaks and negative media attitudes we observed points to the critical importance of conflict mitigation. Compensation schemes have shown limited effectiveness in increasing tolerance (Agarwala et al. 2010; Marino et al. 2016; Bautista et al. 2019), highlighting the need to complement them with effective livestock protection through husbandry practices (Pimenta et al. 2017), tested protection measures (Bruns et al. 2020), and rapid intervention protocols (Van Eeden et al. 2018) to reduce conflict intensity.

Once the effects of recolonization phases and grazing season were accounted for, electoral periods emerged as particularly important in explaining variation in media attitudes. Indeed, proximity to political elections was the variable most strongly associated with negative media attitudes among all variables tested in the 2014–2021 period. Such politicization aligns with patterns in USA, where political affiliation predicts wolf conservation attitudes (Van Eeden et al. 2021), and Germany, where wolves feature prominently in political discourse and campaigns (Clemm von Hohenberg and Hager 2022; Zscheischler and Friedrich 2022). While negative media attitudes during the grazing season reflect news coverage of concrete events (i.e., predations on livestock), the association between negative coverage and election proximity, unrelated to wolf activity, reveals a social and political dimension shaping media narratives. Although increased wolf-related coverage during campaigns could reflect public information efforts, rising negative attitudes cannot be explained by information needs alone. Instead, this aligns with work showing large carnivores deliver political “services”, providing politicians symbols for broader tensions and means to engage interest groups they might otherwise overlook (Chapron and López-Bao 2014), with the risk of management decisions being used to score political points rather than to pursue transparent management objectives (Darimont et al. 2018), a salient concern in the context of the recent downgrading of wolf protection status from ‘strictly protected’ to ‘protected’ under the Habitats Directive (Council of the EU 2025). This politicization appears largely constructed as broad public support with limited polarization has been documented across Europe (Chapron et al. 2025), contrasting with electoral-period media negativity.

## Conclusion

Given the heightened interest during campaigns and the media’s influential role in shaping management (Fernández-Gil et al. 2016), conservation of this controversial species risks being driven more by electoral cycles than by scientific evidence. Hence, fostering coexistence requires acknowledging how the media responds to the wolf’s presence and may shape public perceptions toward it (Enck and Brown 2002). Regularly monitoring media attitude trends can therefore provide valuable insights into the social dimensions of coexistence. From a conservation perspective, transparent, evidence-based communication, and open access to reliable data, for example by encouraging collaboration between journalists and wildlife agencies (Arbieu et al. 2021), may foster a constructive and pluralist dialogue toward long-term tolerance of wolf presence, supporting coexistence as a shared societal goal.

## Supplementary Information

Below is the link to the electronic supplementary material.

Supplementary file1 (PDF 2215 kb)

## Data Availability

The data used in this study are available at the following Zenodo repository: https://doi.org/10.5281/zenodo.15774206.

## Footnotes

1 www.giornalidelpiemonte.it.
