# Supplementary material for: Media attitudes toward wolves reflect recolonization phases, livestock predation peaks, and electoral cycles
Source: Ambio. 2026 Apr 26;55(12):2921–31. doi: 10.1007/s13280-026-02408-9 (PMC13731608; doi:10.1007/s13280-026-02408-9)
Supplement: Supplementary file 1 [file 13280_2026_2408_MOESM1_ESM.pdf]

Supplementary Information: This Supplementary Information has not been peer reviewed.

Title: Media attitudes toward wolves reflect recolonization phases, livestock predation peaks, and electoral cycles

Supplementary Information on Sampling Design of Data Collection and Media Attitude Assessment

We assembled a dataset of 4094 Italian wolf-related news article, partitioned into two complementary subsets based on assessment method. The Manual Dataset comprises articles collected during the LIFE Wolfalps projects (2014–2017 & 2020–2021; n = 1778 total), of which 1622 online news articles were used in subsequent analyses. An additional 156 articles from printed newspapers' archives (www.giornalidelpiemonte.it) were originally collected during the LIFE project periods to broaden coverage of the early sampling years (2014–2016), when online news was comparatively scarce, and were assessed by the 100 readers alongside the online articles. Once the online dataset proved sufficient for the main analyses, these articles were excluded from subsequent analyses to maintain methodological consistency with the Google News-based collection strategy. However, they were retained as supplementary training material for the neural network, where their printed-press format and concentration in the early years provided additional context useful for training. Within the Manual Dataset, 276 articles could not be fully transcribed due to time and logistic constraints. Readers nonetheless assessed these articles based on PDF copies of the web pages, though the article text was not extracted for neural network training. The Automated Dataset covers periods outside the LIFE projects (2018–2020 & 2022–2023; n = 2472). Year 2020 was supplemented using identical methods to address incomplete coverage during the LIFE project period. For 56 paywall-restricted articles, we retained only titles and subtitles. To harmonize both datasets, all headlines (and full texts when necessary) were screened against two inclusion criteria:

1. Relevance to wolves. We kept any articles where wolves featured substantively, even if peripherally (e.g., a news story primarily about the bear population that includes a paragraph on wolves). We discarded cases of rapid misidentification (e.g., an attack quickly reattributed from wolf to dog where the article focuses on the dog breed).
2. Exclusion of non-news content. We omitted pieces treating wolves in symbolic, paleontological, or comparative-behavior contexts unrelated to current events (e.g., why dogs wag but wolves do not).

Application of these inclusion criteria, followed by inspection of the complete dataset and spatial-temporal patterns (Figure S1), reveals that, despite potential search engine algorithm evolution or changes in digital archiving practices over the study period, the consistent sampling strategy yielded continuous trends in data collected across categorized areas with an increase in media coverage linked to the expansion of the species' presence (Figure S1), with a concurrent increasing frequency of national-level news coverage. Finally, we compiled election dates at two scales: (i) national (Italian and European general elections, involving all provinces) and (ii) regional (seven Alpine regions, involving their constituent provinces). Whenever our last observed election preceded 31 December 2023, and no subsequent

election date was defined, we projected the next election by adding the standard five-year interval to the most recent known date.

We assigned the 1778 articles in the Manual Dataset to 100 readers using a stratified random design that balanced four characteristics—sex, age, wolf knowledge, and preexisting attitude—and asked each reader to rate the article's attitude toward wolves. Of these readers, 52 identified as female and 48 as male. Age ranges were under 30 (n = 19), 30–49 (n = 36), 50–69 (n = 37), and 70 or older (n = 8). Self-reported knowledge levels were little-to-none (n = 5), basic-to-intermediate (n = 60) and in-depth (n = 35). Readers' attitudes towards the species on a five-point Likert scale (–2 to +2) fell into neutral (0; n = 12), favorable (+1; n = 44), and strongly favorable (+2; n = 44). We calibrated each reader's workload based on availability to yield exactly four independent ratings per article, ranging from 25 to 200 articles with a median of 100 articles each. With this partitioning, we obtained four attitude assessments for every article in the Manual Dataset, including the 276 "Not Transcribed" articles. In the rare cases of readers failing to record a score, we retained only the valid assessments. Upon further examination, we identified a minority of duplicated articles, occasionally causing more than four assessments per article. During the analysis, we randomly dropped surplus assessments.

We leveraged our manually coded media attitudes to train the automated BERT-based neural network classifier in Python 3 using Tensorflow 2.10.0 (Abadi et al. 2015). Starting from the pre-trained "BERT-Base Uncased" layer, we added a dropout layer, a dense layer, and an output layer corresponding to our media attitude categories. We used a slanted-triangular learning rate schedule, limited the number of epochs, kept the architecture shallow, and centrally truncated any input exceeding the BERT token limit. We replaced the usual cross-entropy loss with an ordinal cross-entropy loss to capture the ordered nature of our Likert scale. We excluded the 276 "Not Transcribed" articles, then partitioned the remaining Manual Dataset by date: articles from 1 May 2015–30 April 2016 (n = 173) became our test dataset; those from 1 May 2020–30 April 2021 (n = 282) our validation dataset; and the other 1047 articles our training dataset. Because BERT-Base Uncased requires English input, we translated all Italian texts using the Excel GOOGLETRANSLATE function. We over-sampled underrepresented media attitude classes to correct class imbalances in training by duplicating texts and assessment modes of articles. We experimented with data augmentation methods, namely SMOTE (Chawla et al. 2002), to avoid direct copies of already present articles finding no performance gain. Once the neural network was trained, a process that consisted of receiving the tokenization of the articles' text as input and providing the articles' reader assessments as output, it produced 12 independent attitude predictions per article in the validation and test sets. Since the predictions of media attitudes obtained with the output layer are probabilistic, they were coded into classes of media attitudes using fine-tuned thresholds. To emulate our four-reader design and dampen stochastic variation, we partitioned each set of 12 predictions into four groups of three, took the mode within each group (breaking ties at random), and thus generated four ratings per article, directly analogous to the Manual Dataset.

We tracked four complementary metrics to improve our classifier's alignment with human assessments. Two metrics derive directly from the 5-point Likert ratings: Mean Squared Error (MSE), the average squared difference between predicted scores and true scores, and Quadratic Weighted Kappa (QWK), a chance-corrected agreement statistic that penalizes larger rating discrepancies more heavily. For both MSE and QWK, we computed two values: *Reader vs. Reader*, comparing each reader's assessment of an article to the other readers' assessments of that same article, thereby quantifying inter-reader consistency; *Neural Network vs. Mode of Readers*, comparing each neural network prediction on an article to the mode of the four human assessments on that same article, thereby quantifying the neural network's consistency with the most likely reader. These two metrics can be derived directly from the confusion matrices obtained for the validation and test sets shown in Figure S2, where better metrics will correspond to higher scores on the diagonals. The remaining two metrics quantify how well the neural network recreates the subdivision into media attitude classes obtained from the human assessments, coded in the underlying normal distribution that creates such subdivisions. Given that a normal distribution is characterized by a mean and a standard deviation, we used the neural network (NN) predictions and the assessments obtained from the mode of the readers (MoR) to compute $\Delta\mu = \mu_{NN} - \mu_{MoR}$, the difference in distribution means, and $\Delta\sigma = \sigma_{NN} - \sigma_{MoR}$, the difference in distribution standard deviations. So, these latter two metrics, $\Delta\mu$ and $\Delta\sigma$, are obtained by treating the MoR distribution as the "gold standard" to be achieved by the neural network's predictions. To estimate μ_{NN}, μ_{MoR}, σ_{NN}, and σ_{MoR} we leveraged a Bayesian hierarchical ordinal regression model:

$$P(\mu, \sigma, \tau_{-1|0}, \tau_{0|1} \mid V_i, \tau_{-2|1}, \tau_{+1|+2}) \propto \prod_{i=1}^{M} C(V_i \mid p(\mu, \sigma)) \times U(\sigma \mid 0.1, 2.5) \times N(\mu \mid 0, 1^2) \times N(\tau_{-1|0} \mid -0.5, 1^2) \times N(\tau_{0|+1} \mid 0.5, 1^2) \quad (S.1)$$

Where $P(\cdot)$ are the posterior distributions of the parameters, M is the number of available article's assessments and V_i their media attitude values. The thresholds $\tau_{k|k+1}$ follow the same logic described in the methodology. Here C is the categorical distribution, for which the probability p are obtained as

$$p_k = \Phi\left(\frac{\tau_{k|k+1} - \mu}{\sigma}\right) - \Phi\left(\frac{\tau_{k-1|k} - \mu}{\sigma}\right) \quad \text{for} \quad k \in (-2, -1, 0, +1, +2) \quad (S.2)$$

with $\Phi(\cdot)$ being the standard normal cumulative distribution function and considering the constraint $\tau_{k-1|k} \leq \tau_{k|k+1}$ where, if $k = -2$, then $\tau_{k-1|k} = -\infty$, while if $k = 2$, then $\tau_{k|k+1} = \infty$.

By jointly monitoring these four metrics, we ensured that our classifier produced both accurate individual ratings and a faithful overall distribution of media attitudes. Relying solely on metrics that highlight the correctness of assessments (MSE and QWK) would have let the neutral category (0, the modal media attitude) dominate, artificially inflating agreement simply by predicting the most likely outcome. Conversely, focusing only on the reliability of the media attitude distribution ($\Delta\mu$ and $\Delta\sigma$) would have optimized for matching class frequencies without guaranteeing that any single article's score was correct.

Table S1 reports the metrics obtained by averaging over the four assessments of the neural network. We judged the neural network as reliable on the validation set when these two conditions occurred for the validation set: 1) its MSE was lower, and its QWK higher, in the *Neural Network vs. Mode of Readers* comparison than in the *Reader vs. Reader* comparison; 2) $\Delta\mu$ and $\Delta\sigma$ were negligible and all estimates of μ_{NN} and σ_{NN} laid within the 95 % CI of the μ_{MoR} and σ_{MoR} estimates. These criteria also guided our final hyperparameter choices, fixing their values when performance reached these standards. When we applied the trained neural network to the held-out test set, we observed a $\Delta\mu \lesssim 0.1$, indicating a very slight positive bias relative to the manual classifications, but all μ_{NN} estimates remained within the 95 % credible interval of μ_{MoR}, so this bias relevance is unclear. Meanwhile, all other metrics showed no criticality: the *Neural Network vs. Mode of Readers* MSE and QWK both improved on the test set (more than doubling their relative gains over *Reader vs. Reader* baselines); σ_{NN} increased appropriately from 1.13 to 1.29 matching the shift in σ_{MoR}, demonstrating remarkable ability to adapt class proportions to new data. In sum, our four-metric framework confirmed that the BERT-based classifier replicates human consensus more reliably than individual readers replicate one another while preserving the full range of media attitudes by showing no systematic bias or undue compression/expansion of the media attitude distribution. This validates its use for extending our media attitude analysis to the larger Automated Dataset, though effects smaller than 0.1 in $\Delta\mu$ should be interpreted with caution.

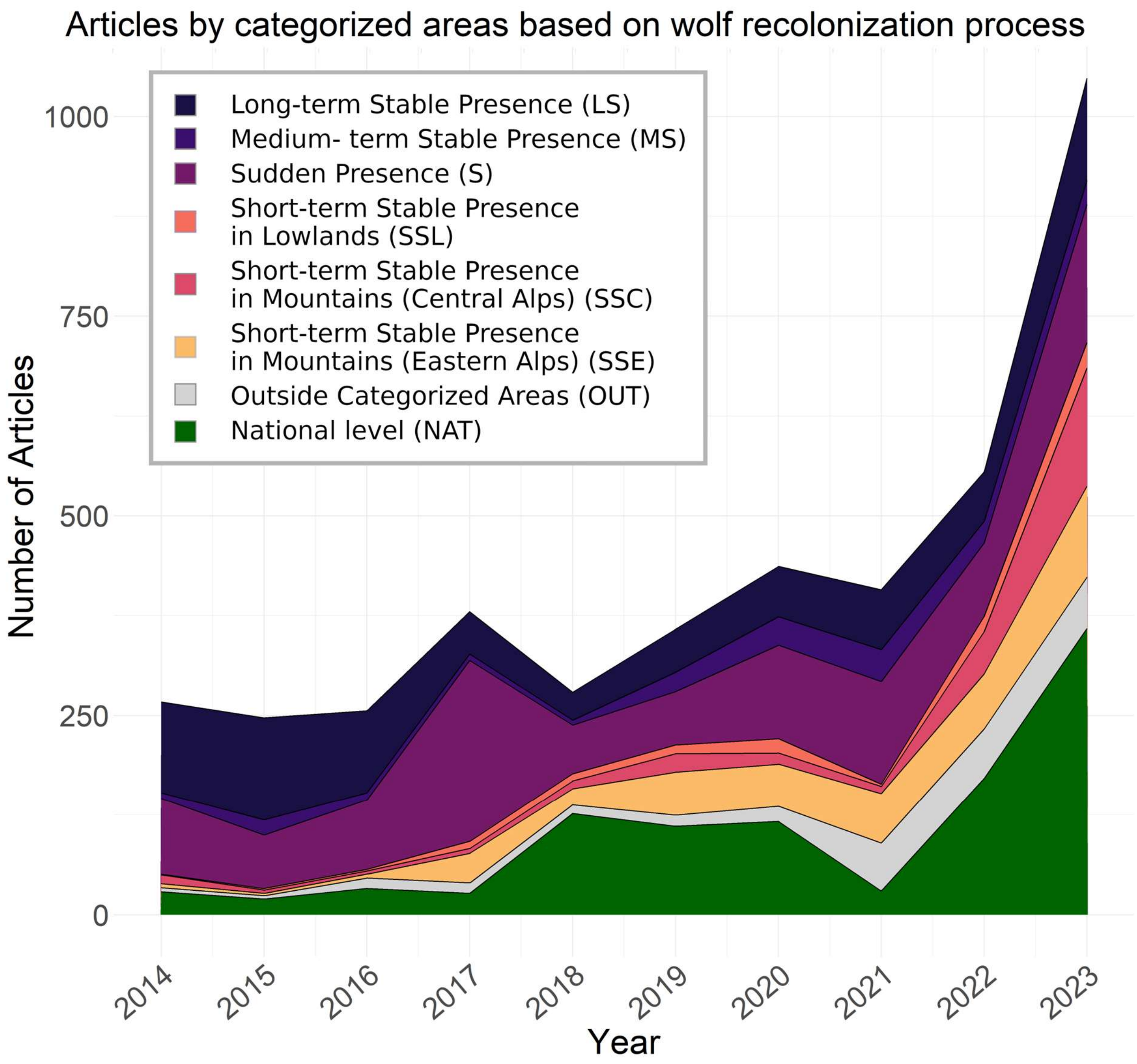

Figure S1. Annual volume of wolf-related news articles, 2014–2023, broken down by spatial attribution. In 2022, and especially in 2023, the cumulative contribution of the three short-term stable presence areas, Mountains (Eastern Alps) (SSE), Mountains (Central Alps) (SSC), and Lowlands (SSL), grow rapidly, signaling the emergence of local media attention in these areas.

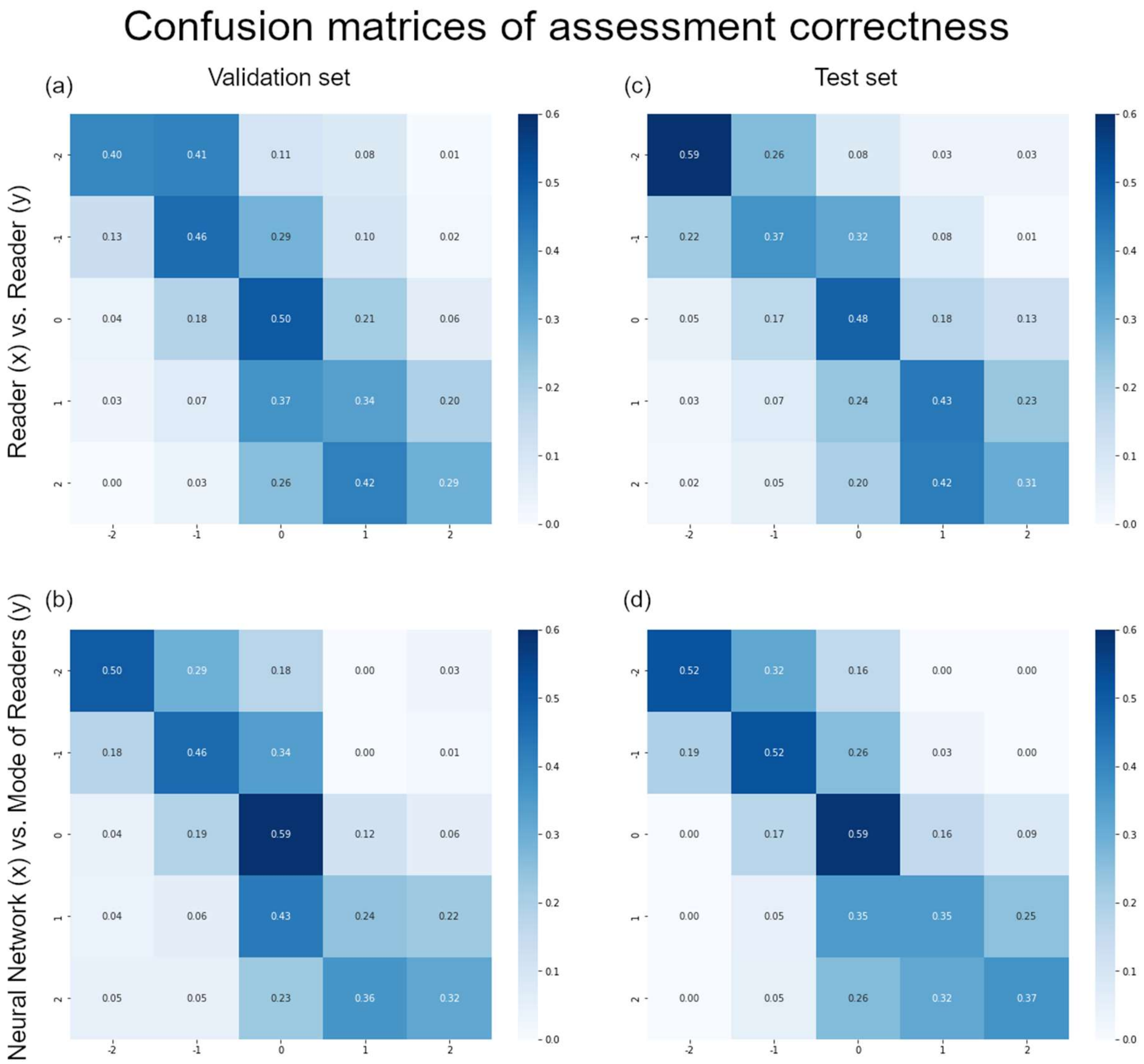

Figure S2. Normalized confusion matrices comparing *Reader vs. Reader* (*a* and *c*) and *Neural Network v.s Mode of Readers* (*b* and *d*) assessments. Both when confronting the two matrices obtained for the validation set (*a* and *b*) and for the test set (*c* and *d*), the *Neural Network vs. Mode of Readers* confusion matrices show a more densely populated diagonal than the *Reader vs. Reader* matrices, reflecting the improvement in MSE and QWK shown in Table S1.

Metrics used to monitor neural network performance										
Assessments correctness	MSE		QWK		Reliability of media attitude distribution	μ		σ		
	Val.	Test	Val.	Test		Val.	Test	Val.	Test
Neural Network vs. Mode of Readers	0.96	0.85	0.582	0.692	Neural Network normal distribution ($\overline{\mu_{NN}}$ and $\overline{\sigma_{NN}}$)	-0.133 (-0.116; -0.212; -0.213; 0.010)	-0.019 (0.050; -0.055; -0.109; 0.038)	1.13 (1.24; 1.05; 1.07; 1.15)	1.29 (1.29; 1.23; 1.27; 1.37)
Reader vs. Reader	1.12	1.19	0.559	0.616	Mode of Readers normal distribution (μ_{MoR} and σ_{MoR})	-0.141 [-0.308; 0.036]	-0.106 [-0.35; 0.135]	1.16 [1.04; 1.29]	1.33 [1.16; 1.53]
Relative difference	-0.16	-0.34	0.024	0.077	Relative difference ($\Delta\mu$ and $\Delta\sigma$)	0.008	0.087	-0.03	-0.05

Table S1. The four performance metrics on both validation and test sets. On the left are reported the measures regarding correctness of assessments, Mean Squared Error (MSE) and Quadratic Weighted Kappa (QWK), each computed twice: once comparing *Neural Network vs. Mode of Readers* and once comparing *Reader vs. Reader*. On the right are shown the distribution-reliability measures obtained from the differences in mean (μ) and standard deviation (σ) between the parameters that characterize the neural network's assessments normal distribution (*NN*, obtained by averaging the four median estimates obtained with the model) and that of the mode of the readers assessments (*MoR*). For all *NN* estimates, all four median estimates fall within the 95 % credible interval of the corresponding mode of the readers metric, where we display such interval in square brackets.

Supplementary Information on the Bayesian model

Similarly to what shown in equation $S.1$ and recalling the parameters described in the methodology—the means α_X with X representing a spatial attribution LS, MS, S, SSL, SSC, SSE, NAT, OUT, the slope of the linear temporal trend β, the parameters of the sinusoidal function γ_{Days} and γ_{Ampl}, and the intensity of the election proximity ψ—we obtained their posterior distributions $P(\cdot)$ as:

$$P(\alpha_X, \gamma_{Ampl}, \gamma_{Days}, \beta, \psi, \sigma, \tau_{-1|0}, \tau_{0|+1} \mid V_i, \tau_{-2|-1}, \tau_{+1|+2}) \propto \prod_{i=1}^{M} C(V_i \mid p_i(\mu_i, \sigma)) \times U(\sigma \mid 0.1, 2.5) \times N(\tau_{-1|0} \mid -0.5, 1^2) \times N(\tau_{0|+1} \mid 0.5, 1^2) \times N(\gamma_{Days} \mid 0, 200^2) \times U(\gamma_{Ampl} \mid -1, 1) \times \prod_{X \in Cat.Ar} (\alpha_X \mid 0, 1^2) \times N(\beta \mid 0, 1^2) \times N(\psi \mid 0, 0.5^2) \quad (S.3)$$

Where C is the same categorical distribution shown in equation $S.2$ with the only change being the replacement of μ with μ_i, where μ_i is obtained as shown in the methodology of the main paper. We implemented our hierarchical Bayesian model in R using JAGS. Table S2 reports the posterior medians and 95 % CI for all parameters not shown in Table 1. We fitted the hierarchical model three times: once on the full 2014–2023 dataset, and separately on two non-overlapping time intervals, Period A (2014–2021) and Period B (2022–2023). We chose Period B because it captures both the recent wolf recolonization in three categorized areas and the concurrent surge in media coverage (Figure S1), thereby defining the interval during which recolonization's impact on media attitudes is most detectable.

The slope of the temporal trend (β) appears small or insignificant depending on the dataset used to obtain it and does not appear to match the actual trend of media attitudes. Figure S3 visualizes this trend over 2014–2023 for each spatial attribution: the solid line shows the trend obtained using the posterior median slope β, with its 95 % credible band, while the overlaid boxplot displays the empirically estimated mean media attitude for each year. To generate those yearly means, we held all parameters except the year-and-space mean α_X fixed at their overall posterior medians (β, γ_{Days}, γ_{Ampl}, ψ, $\tau_{-1|0}$, and $\tau_{0|+1}$ from the full 2014–2023 fit), and re-estimated only the α_X terms. Regardless of the intensity of the linear trend of media attitudes, the year-by-year box plots reveal deviations from strict linearity: several annual 95 % CI do not overlap the trend's credible band, showing that media attitudes can fluctuate more sharply than a simple linear model would predict.

We selected $T = 180$ days through an informed sensitivity analysis guided by both empirical model behavior and substantive considerations about electoral discourse dynamics. Empirically, we fit our model repeatedly with T ranging from 30 to 360 days (using both the 2014–2021 and 2014–2023 datasets) and examined how the resulting ψ estimates changed (Figure S4). The estimates stabilized near

$T = 180$ days for the 2014–2021 dataset, and their rate of change decreased near that value for the 2014–2023 dataset, indicating estimate stability. Substantively, a half-life of six months is consistent with how electoral interest typically evolves: public and media attention to elections realistically intensifies only within the final year of a campaign cycle, making half-lives much shorter (30 days) or much longer (400 days) implausible. A value of $T = 180$ days also avoids a numerical issue that arises with larger half-lives. When T is set too high (e.g., 400 days), the decay term $(0.5)^{d_i\,/\,T}$ remains non-negligible even when the next election is nearly three years away (~ 1000 days). This persistent baseline offset can introduce compensatory shifts in the spatial mean parameters α_X and cause mirroring artifacts in the MCMC chains. With $T = 180$ days, the decay term effectively vanishes for large elect$_i$, so inference is stable and α_X is not inflated artificially.

We verified that our model does not systematically bias the election-effect parameter ψ toward negative values by re-estimating ψ using deliberately mis-timed election dates. Specifically, we shifted all election dates once one year forward and once one year backward. For the 2014–2021 dataset, where with the actual election dates we had $\psi = -0.449$, we obtained $\psi_{forward} = -0.048$ (95% CI: [-0.133, 0.041]) and $\psi_{backward} = -0.022$ (95% CI: [-0.131, 0.080]). For the 2014–2023 dataset, where with the actual election dates we had $\psi = -0.114$, we obtained $\psi_{forward} = -0.109$ (95% CI: [-0.176, -0.045]) and $\psi_{backward} = -0.156$ (95% CI: [-0.223, -0.089]). When using fabricated dates on the 2014–2021 data, the 95 % CI both include zero, indicating that the strong negative ψ observed with the true dates does not reappear under date shifts and hence is not an artifact of model bias. In contrast, the 2014–2023 dataset continues to yield a negative ψ even with shifted dates. We attribute this to our artificial placement of a "pseudo-election" in the second half of 2023: shifting once Italy's 25 September 2022 vote to 25 September 2023 and once Europe's 6 June 2024 vote to 6 June 2023 forces the model to explain the observed downturn in media attitudes occurring in 2023 by estimating a negative election effect.

Finally, we verified that our hierarchical Bayesian model can accurately recover known parameter values by generating a synthetic dataset of 10 000 article attitude observations. Drawing uniformly, we assigned to each article a spatial label from a set of eight, a publication year from eight consecutive years, a day-of-year from [1, 365], and a days-to-next-election uniformly from [1, 1350] (being 1350 the maximum observed in our real data). We then simulated these media attitudes using parameter values chosen to be similar to those estimated from our 2014–2021 and 2014–2023 fits. Table S3 compares the true values to the posterior medians and 95 % CI recovered by JAGS: in every case the credible interval contains the true value, demonstrating successful parameter recovery. Finally, we performed posterior-predictive checks on the fits to the 2014–2023, 2014–2021 and 2022–2023 datasets (Figure S5) to confirm that our priors do not unduly constrain the posteriors, and we inspected the MCMC chains (Figure S6, S7, and S8) to ensure adequate mixing and verify that our burn-in of 5000 iterations (without thinning) was sufficient to eliminate dependence on initial values.

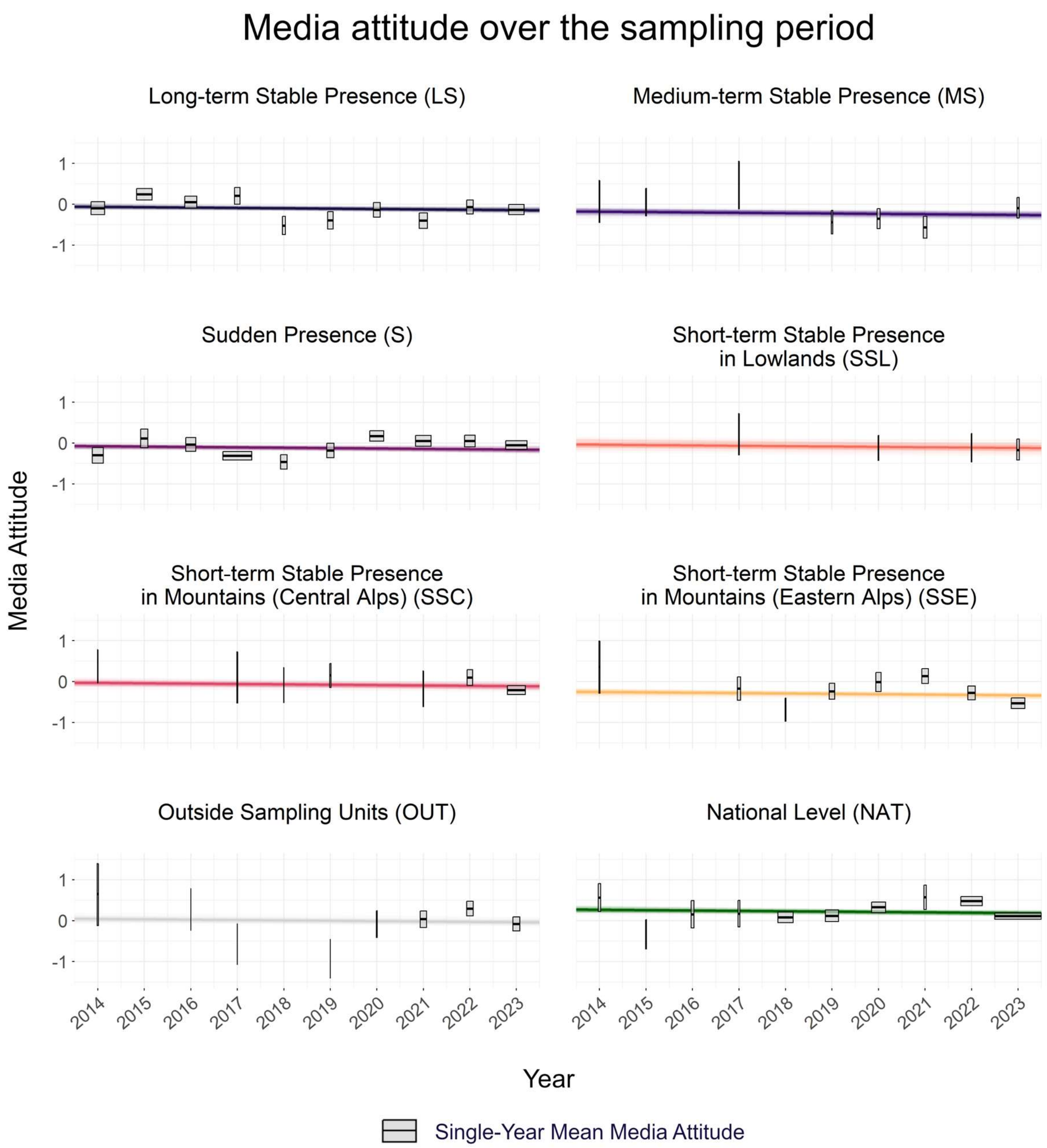

Figure S3. The variation of the means over time, estimated as a linear relationship for the 2014–2023 dataset, divided by spatial attribution. The bands surrounding the main trends are the result of sampling 100 times the 95% CIs of the parameters' posterior distributions. For years in which one of these subsets of data had more than 20 article assessments we estimated the yearly means, whose 95% CIs are shown in the box plots, while fixing the other parameters at their median estimates. The width of each box plot is proportional to the number of articles for that specific year and spatial attribution.

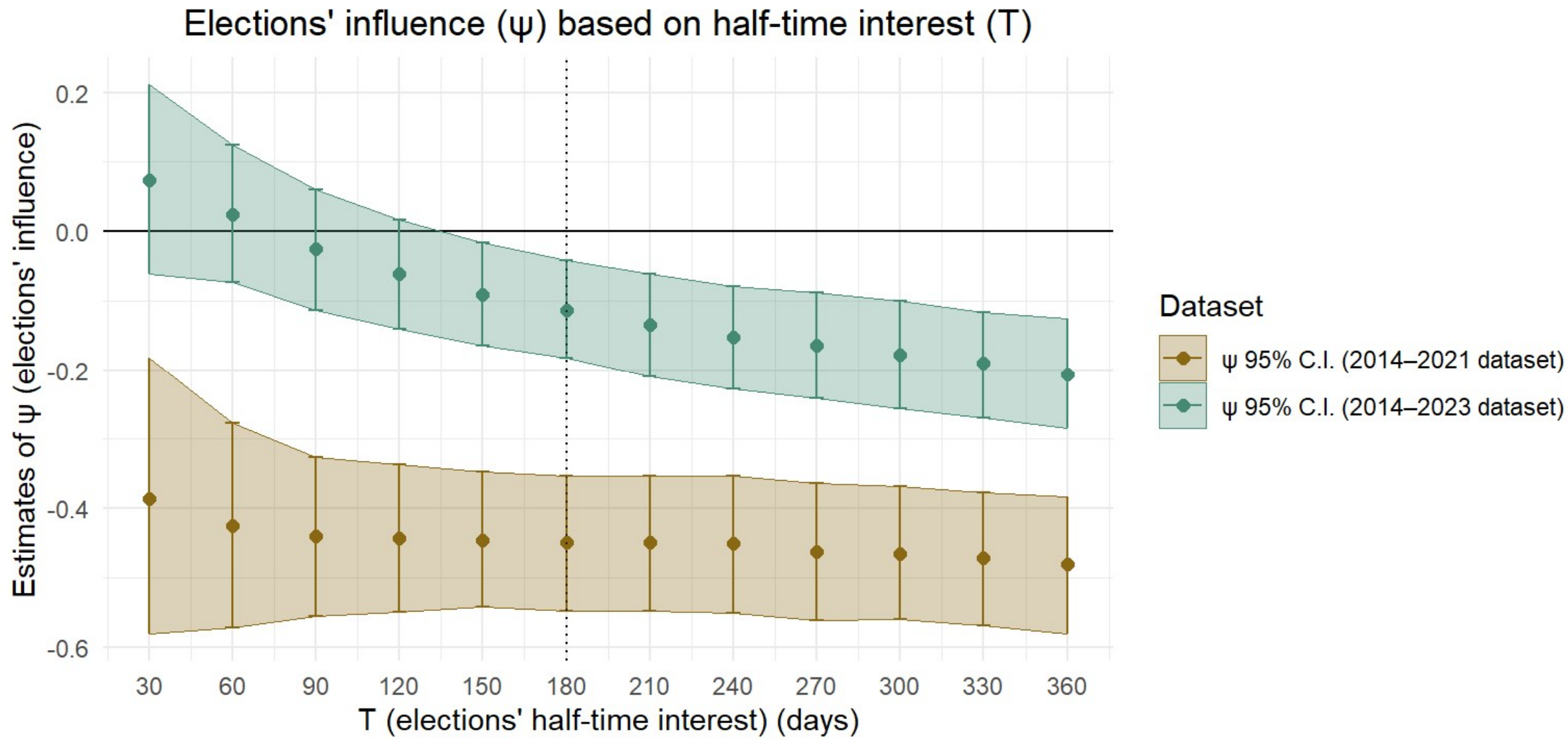

Figure S4. The different estimates of ψ (the influence of proximity to elections) and relative 95% CIs obtained with values of T (the half-life elections interest) going from 30 days to 360 days. We can see how for the 2014–2021 dataset all estimates of the CIs do not intercept 0 and hence consistently show a negative effect linked to elections proximity, while for the 2014–2023 dataset only results with $T \geq 150$ do not intersect the 0. The dotted line at $T = 180$ highlights the value of T chosen and the corresponding estimates of ψ.

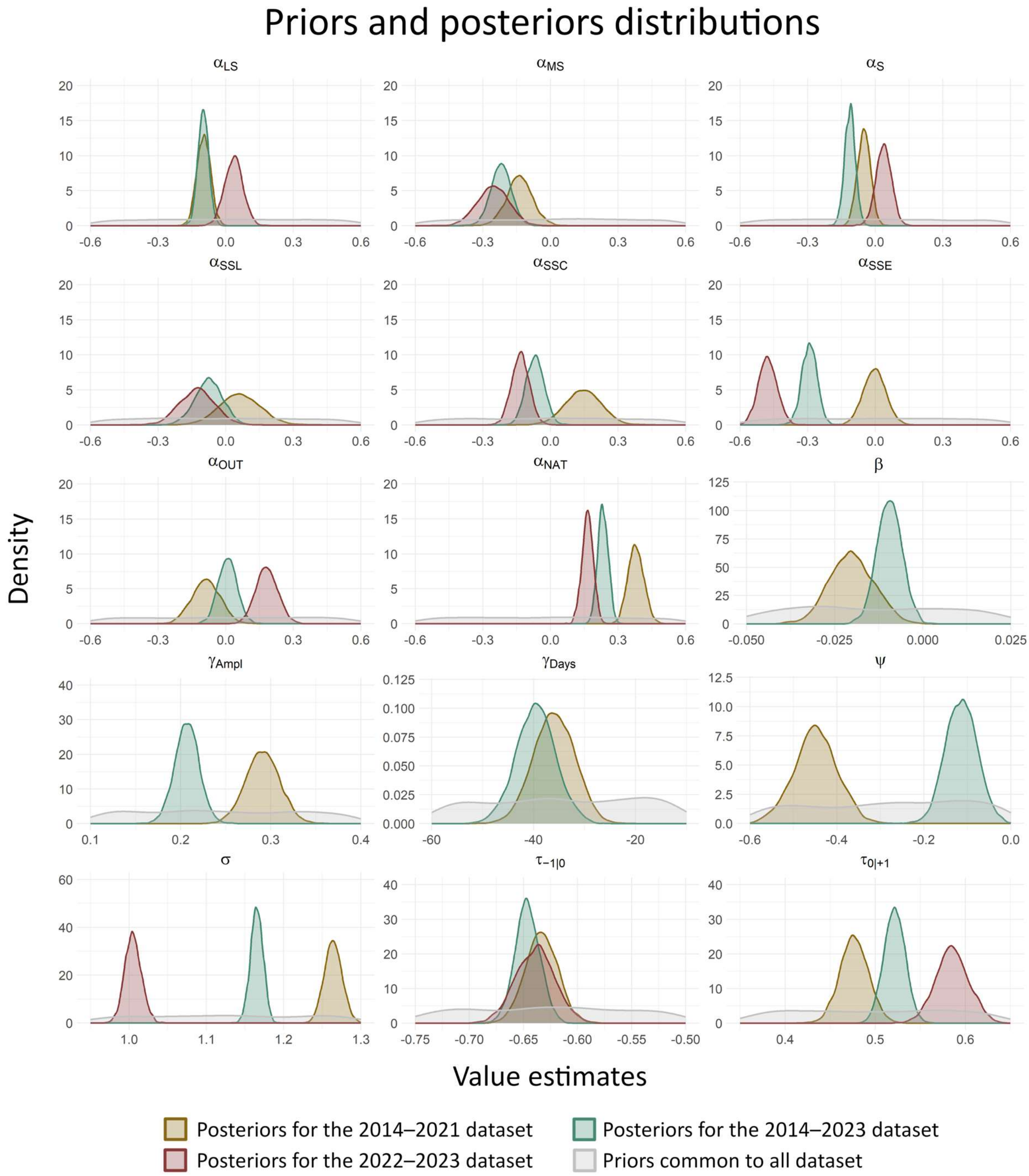

Figure S5. Posterior distributions obtained when running the Bayesian model with the 2014–2021, the 2014–2023, and 2022–2023 datasets compared to the prior distributions used for all the parameters estimated by the model. All posterior distributions are within relatively flat regions of their prior distributions.

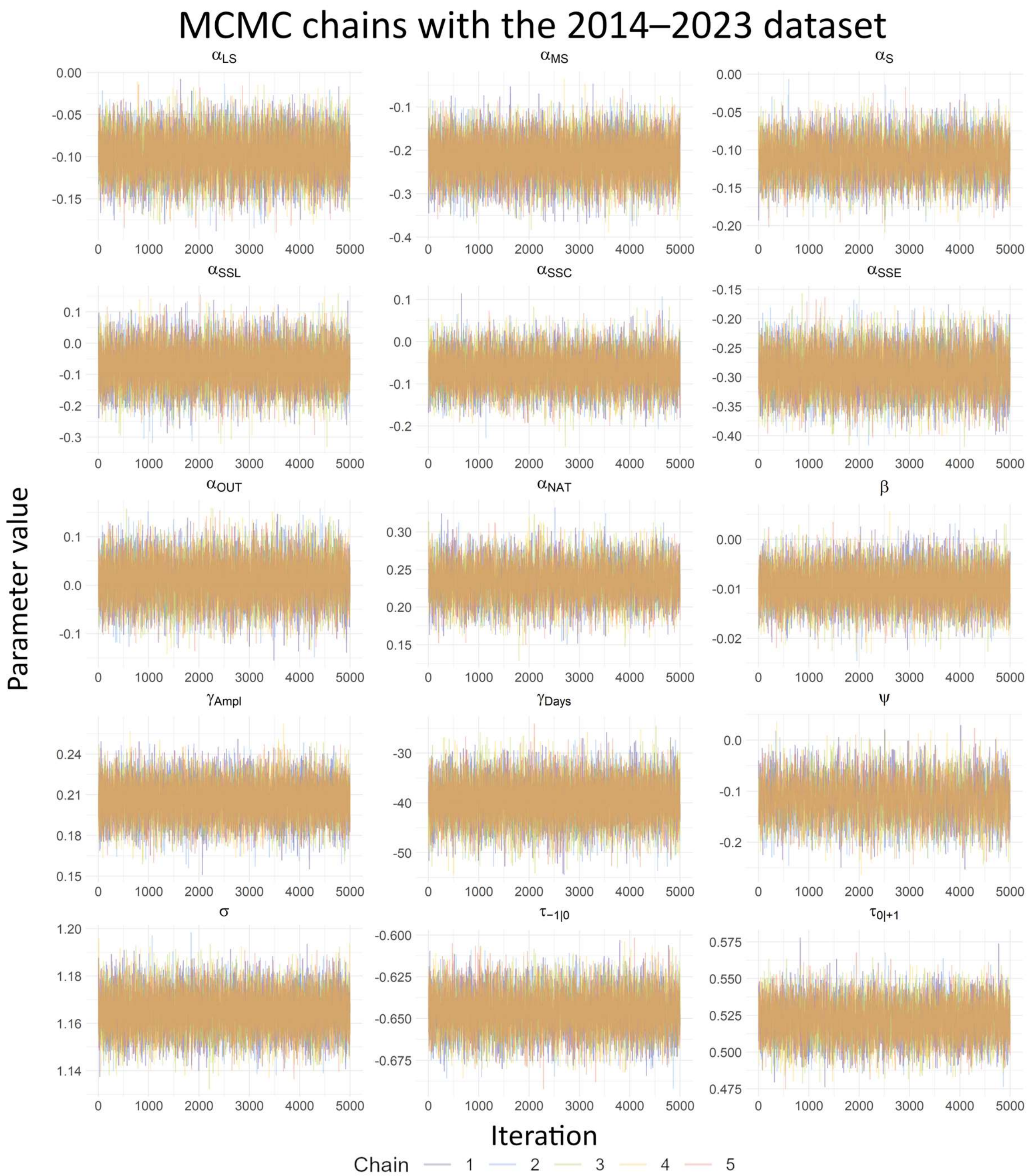

Figure S6. The 5000 iterations of the Markov Chain Monte Carlo (MCMC) chains we obtained when running the Bayesian model with the 2014–2023 dataset. All MCMC chains for the parameters do not show wandering behavior.

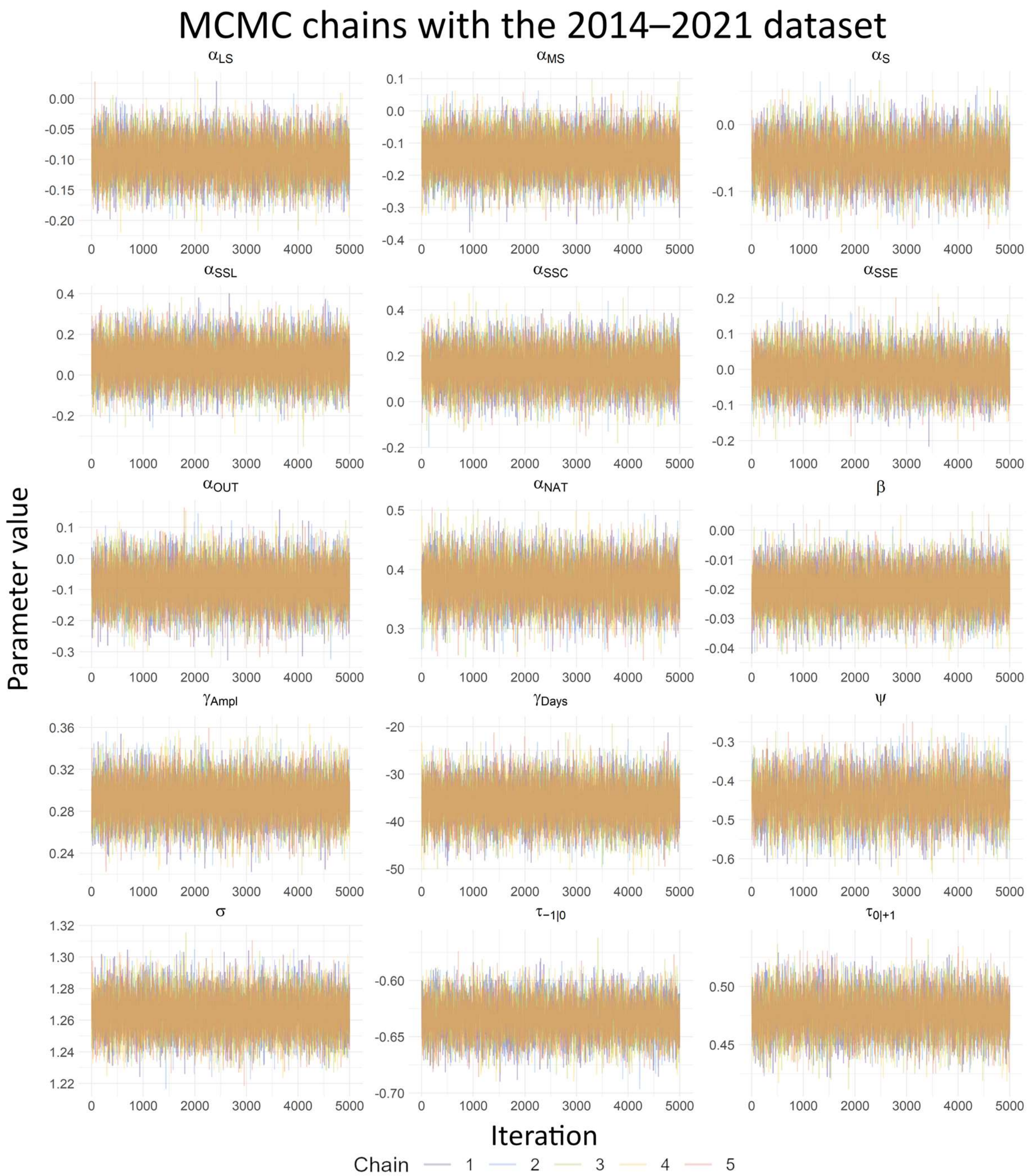

Figure S7. The 5000 iterations of the Markov Chain Monte Carlo (MCMC) chains we obtained when running the Bayesian model with the 2014–2021 dataset. All MCMC chains for the parameters do not show wandering behavior.

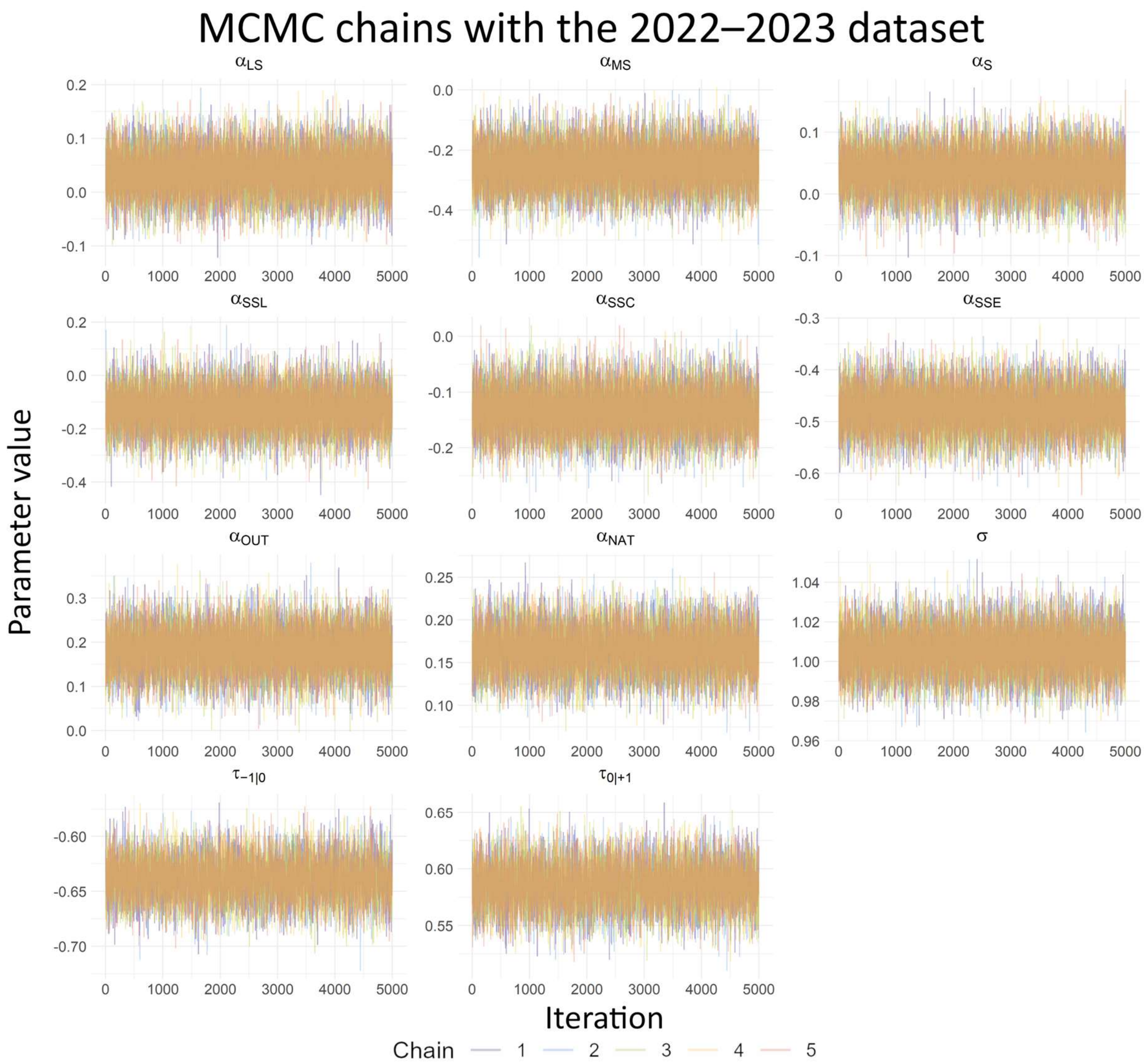

Figure S8. The 5000 iterations of the Markov Chain Monte Carlo (MCMC) chains we obtained when running the Bayesian model with the 2022–2023 dataset. All MCMC chains for the parameters do not show wandering behavior.

Parameters estimated with the Bayesian model not used to test hypothesis					
Parameters			Median and 95% CI		
			2014–2023<br>entire dataset	2014–2021<br>Period A	2022–2023<br>Period B
Mean of local media attitude outside the categorized areas		α_{OUT}	-0.087<br>[-0.208, 0.033]	0.008<br>[-0.072, 0.090]	0.182<br>[0.089, 0.279]
Standard deviation		σ	1.26<br>[1.24, 1.29]	1.17<br>[1.15, 1.18]	0.99<br>[0.97, 1.02]
Thresholds between media attitude classes	between "-1" and "0"	$\tau_{-1\|0}$	-0.63<br>[-0.66, -0.60]	-0.65<br>[-0.67, -0.62]	-0.63<br>[-0.65, -0.60]
	between "0" and "+1"	$\tau_{0\|+1}$	0.48<br>[0.47, 0.51]	0.52<br>[0.49, 0.54]	0.59<br>[0.55, 0.62]

Table S2. The median and 95% CI of the parameters estimated by the Bayesian model for the different datasets not shown in Table 1.

Capability of the model to recover the parameters				
Parameter	Value used to Simulate Data	Recovering the Parameter		
		Prior	Median	95% CI
α_{LS}	0.5	$N(0, 1^2)$	-0.53	[-0.61, -0.46]
α_{MS}	0.5	$N(0, 1^2)$	0.48	[0.40, 0.55]
α_{S}	0	$N(0, 1^2)$	0.02	[-0.06, 0.09]
α_{SSL}	-1	$N(0, 1^2)$	-0.97	[-1.05, -0.90]
α_{SSC}	1	$N(0, 1^2)$	0.99	[0.91, 1.06]
α_{SSE}	-0.2	$N(0, 1^2)$	-0.21	[-0.28, -0.13]
α_{OUT}	0.2	$N(0, 1^2)$	0.19	[0.11, 0.26]
α_{NAT}	0	$N(0, 1^2)$	0.06	[-0.01, 0.13]
β	$-1.00 \bullet 10^{-2}$	$N(0, 1^2)$	$-0.99 \bullet 10^{-2}$	[$-2.06 \bullet 10^{-2}$, $0.10 \bullet 10^{-2}$]
γ_{Ampl}	0.2	$U(-1, 1)$	0.174	[0.138, 0.209]
γ_{Days}	40	$N(0, 200^2)$	46.6	[34.9, 58.4]
ψ	-0.4	$N(0, 0.5^2)$	-0.367	[-0.471, -0.263]
σ	1.2	$U(0.1, 2.5)$	1.20	[1.18, 1.22]
$\tau_{-1\|0}$	-0.80	$N(-0.5, 1^2)$	-0.80	[-0.82, -0.77]
$\tau_{0\|+1}$	0.60	$N(0.5, 1^2)$	0.60	[0.57, 0.63]

Notes: * in the model the sinusoidal pattern was formulated as a sine wave, which we later transposed to a cosine with the due transposition (Table 1) to better highlight the meaning of the variables.

Table S3. For each parameter estimated by the Bayesian model, we report the values used while simulating the dataset, the priors set for each parameter, which correspond to the priors we used in the model, the recovered parameter medians, and their 95% CIs. We rounded the latter two to the third significant digit, grouping them by parameter type. The close alignment between the simulated and recovered values demonstrates the model's effectiveness in accurately estimating the parameters. Note that in the model the sinusoidal pattern was formulated as a sine wave, which we later transposed to a cosine with the due transposition (Table 1) to better highlight the meaning of the variables.

Supplementary Information References

Abadi, M., A. Agarwal, P. Barham, E. Brevdo, Z. Chen, C. Citro, G. S. Corrado, A. Davis, et al. 2015. TensorFlow: Large-scale machine learning on heterogeneous systems. Mountain View, CA: Tensorflow.

Chawla, N. V., K. W. Bowyer, L. O. Hall, and W. P. Kegelmeyer. 2002. SMOTE: synthetic minority over-sampling technique. *Journal of artificial intelligence research* 16: 321–357.
